# Caesarean section: epidemiology and indications at General Provincial Hospital of Kananga

**DOI:** 10.11604/pamj.2022.42.317.34970

**Published:** 2022-08-29

**Authors:** Antoine Tshimbundu Kayembe, Sylvain Mulumba Kapuku

**Affiliations:** 1 Department of Gynaecology and Obstetrics, Faculty of Medicine, University Notre-Dame of Kasayi, Central Kasaï, Democratic Republic of the Congo

**Keywords:** Caesarean section, epidemiology, indications, Provincial General Hospital, Kananga

## Abstract

The aim of our study is to describe the epidemiological profile and indications for caesarean section at the maternity ward of the Provincial General Hospital of Kananga from January 1^st^ 2014 to December 31^st^ 2020. This is a descriptive study of a series of cases from the medical records of cesarean patients and based on non-probabilistic sampling of suitability for cases selection. The sample of our study is composed of 1395 cases. The frequency of caesarean section is 16.10% with an annual average of 199.30 (SD 18.20) cases per year, the average age of patients is 25.39 years (SD 1.23), the primiparity is the most affected with 27.38 %, hemorrhagic placenta previa is the most common indication in 26.86%, maternal complications is present in 46.73% and the maternal mortality rate due to cesarean section is 531 per 100.000 live births. The practice of caesarean section constitutes a real problem and our results can serve as a basis for in-depth studies on indications and maternal complications of caesarean section in order to reduce the risks associated with the practice of caesarean section in our environment.

## Introduction

The caesarean section is an artificial delivery technique that allows fetal extraction after surgical opening of the uterus. Its purpose is to save the newborn and the mother in situations of fetal distress or dystocia [1, 2]. The frequency of the caesarean section has continued to increase in recent decades, but this increase differs enormously from one country to another and in the same environment, from one medical institution to another [3, 4]. The reasons are multifactorial and not always defensible: changes in maternal characteristics and professional practice styles, increased pressure for malpractice, as well as economic, organizational, social and cultural factors have all been implicated [5, 6]. To these are added the advances in surgery, anesthesiology and blood transfusion ensuring a certain safety in the realization of the cesarean section [7]. In low-resource countries, the frequency of caesarean sections increased from 1.9% to 6.1% [3].

In the Democratic Republic of Congo (DRC), the latest Demographic and Health Surveys report that caesarean section rates rose from 4% in 2007 [8] to 5% in 2013 [9]. As the frequency of caesarean sections increases, the relative importance of the various indications changes. Although rates of elective caesareans are increasingly high in developed countries [10], emergency caesareans are still too frequently performed in sub-Saharan Africa [11, 12] despite the deleterious effect on fetal and maternal prognosis by significantly increasing the related morbidity and mortality [13]. The lack of epidemiological data on the caesarean section in our milieu prompted us to conduct this study, the aim of which is to describe the epidemiological profile and indication of caesarean sections at the maternity of Provincial General Hospital (PGH) of Kananga from January 1^st^ 2014 to December 31^st^ 2020.

## Methods

**Study design and setting:** this is a descriptive study of a series of caesarean section cases recorded at the maternity ward of the PGH of Kananga from January 1^st^ 2014 to December 31^st^ 2020. The PGH maternity ward was chosen because its situation as the second provincial reference hospital for cases, the presence of trained and experienced staff, and the high attendance of patients who trust its staff.

**Study population:** we used the medical files of patients aged between 16 and 45 years old who underwent caesarean section recorded at the maternity unit of PGH of Kananga from January 1^st^ 2014 to December 31^st^ 2020.

**Sampling:** our sampling is non-probabilistic of convenience. The sample size was determined by the limitation of our study in time and space. The following criteria allowed us to include the patients in the study: patients aged between 16 and 45 years old, having undergone caesarean section at the maternity of PGH of Kananga from January 1^st^ 2014 to December 31^st^ 2020 and whose medical file was full. Patients who did not meet these inclusion criteria and incomplete medical files were excluded.

**Collection of data:** data were collected from operating room registers, maternity registers, patient medical files of the maternity of PGH of Kananga and the data collection record. The variables of our study are: year of study, age of patients, parity, indication of cesarean section, complications of cesarean section, treatment initiated and post-therapeutic evolution.

**Statistical analysis:** data were analyzed using Statistical Package for Social Sciences (SPSS) software version 20. We used the average (SD) to present the quantitative variables and the proportion to present the qualitative variables.

**Ethical considerations:** principles of medical ethics and documentary studies rules have been respected; the data were collected confidentially and treated anonymously.

## Results

**Figure 1:**
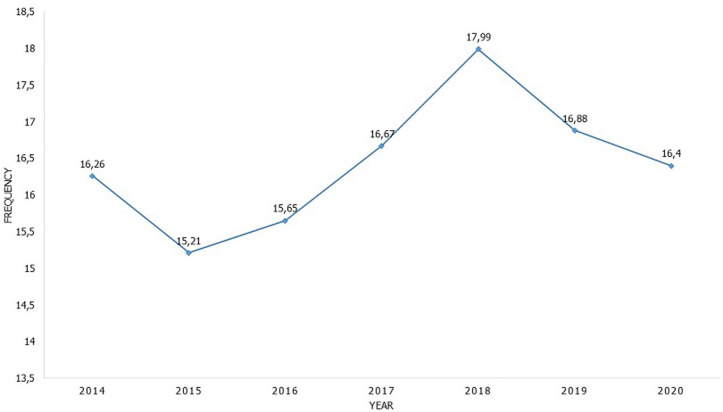
frequency’s evolution of caesarean-section during our study’s period

**Frequency of caesarean section:** we recorded 1395 caesareans out of 8668 deliveries at the maternity unit of PGH of Kananga during the study period, i.e. a hospital frequency of caesareans of 16.10%, the evolution of which during the period of our study varied from 16.26% in 2014 to 16.41% in 2020 with a peak of 17.99% in 2018 and a low frequency of 15.21% in 2015. The average caesarean section case is 199.30 (SD 18.20) cases per year (Figure 1).

**Age and parity of patients:** the age group most affected by caesarean section is between 16 and 20 years old with 433 cases or 31.02% and the average age of patients is 25.39 years (SD 1.23). Primiparity was encountered in 382 cases or 27.38%, pauciparity in 347 cases or 24.87%, multiparity in 363 cases or 26.02% and grand multiparity in 303 cases or 21.72% (Table 1).

**Table 1:** age and parity of patients who have had caesareans

Age range	N=1395	%
16-20	433	31.02
21-25	375	26.86
26-30	209	15.00
31-35	140	10.00
36-40	153	11.00
41-45	85	6.10
**Parity**		
Primiparity	382	27.38
Pauciparity	347	24.87
Multiparity	363	26.02
Grand multiparity	303	21.72

**Indications and complications of caesarean section:** hemorrhagic placenta previa is encountered in 375 cases or 26.86%, foeto-pelvic disproportion in 192 cases or 13.85%, dynamic dystocia complicated by acute fetal distress in 209 cases or 14.95%, uterine rupture in 185 cases or 13.29%, the pelvis narrowed in 93 cases or 6.64%, prolapse of the beating cord in 100 cases or 7.17%, transverse presentation in 97 cases or 6.92%, fetal malposition in 97 cases or 6.92% and other indications in 47 cases or 3.40%. 65.0% of caesarean sections were performed in a context of obstetric emergencies from the outset, 40.50% after attempted vaginal delivery and 4.50% in a scheduled or elective manner. As for complications of caesarean section, they are present in 610 cases or 46.73% (Table 2).

**Table 2:** the indications and complications of caesarean section

Indications of caesarean-section	N=1395	%
Hemorrhagic placenta previa	375	26.86
Foeto-pelvic disproportion	192	13.85
Dynamic dystocia complicated by acute fetal distress	209	14.95
Uterine rupture	185	13.29
Narrowed pelvis	93	6.64
Flapping cord prolapse	100	7.17
Transversal presentation	97	6.92
Fetal malposition	97	6.92
Others	47	3.40
**Maternal complications of caesarean section**		
Present	610	46.73
Absent	785	56.27
**Post-therapeutic evolution**		
Maternal death	45	0.53103

**Initiated treatment and post-therapeutic evolution:** blood transfusion is encountered in 204 cases or 33.44%, surgical repair in 301 cases or 49.34% and other treatments in 105 cases or 17.21%. As for the post-treatment evolution, it is characterized by maternal death in 45 cases corresponding to 531 maternal deaths per 100.000 live births (Table 2).

## Discussion

The hospital frequency of caesarean section was 16.10% at the maternity ward of the PGH of Kananga. Its evolution during the period of our study varied from 16.23% in 2014 to 15.19% in 2020. Our frequency of caesarean section is around three times that observed in the general population in the DRC [9]. It is superior to that of kinenkinda *et al*.in Lubumbashi [14], that of Chu *et al*. in the 4 health districts of 3 African countries (DRC, Burundi and Sierra Leone) [11] and those of Cavallaro *et al*. in sub-Saharan Africa [15] which are respectively 10.65%, 6.2% and varying from 5 to 10%. Several factors would justify the low rates of caesarean section such as: the underqualification of the personnel working at the level of the prenatal consultations [12, 16], the lack of access to this surgical act because the majority of qualified doctors live in urban areas and the Most hospitals have insufficient infrastructure for emergency surgical care [14, 15, 17]. This was not the case in our environment. Our frequency is much lower than those of many other African studies including those of Lokomba *et al*. in Kinshasa [18], of Ouedraogo *et al*. in Ouagadougou [19], of Bokossa *et al*. in Abidjan [20], of Mbungu *et al*. in Kinshasa [21] and of Foumane *et al*. in Yaoundé [13] which are respectively 28.50%, 30.30%, 31.03%, 31.20% and 19.70%. It should be noted that in sub-Saharan Africa, where the frequency of population caesareans was still very low, in recent years we have seen an increase in referral hospitals. In general, the frequency of caesarean sections is high in hospitals with a very high complexity score, in private maternities or those belonging to non-governmental organizations and in establishments where there is a staff motivation system [22]. Which was not the case in our milieu. According to the WHO, the frequency of caesarean deliveries within a health care facility varies considerably depending on the composition of the obstetrical populations it serves, its capacities and resources, and its protocols for clinical care. Because of these differences, the recommended caesarean section rate at the population level cannot be considered the ideal rate at the hospital level [23]. According to the most recent estimates, caesarean sections should represent 18.6% of deliveries on average, with extremes of 6.0% to 27.2% respectively in the least and most developed regions [23]. Although our frequency is within this acceptable range, the need is not only the frequency figure for caesareans but also the improvement of the fetal prognosis while minimizing the maternal risk during caesarean sections. This was the case in our study setting.

The annual frequency of caesarean sections varied during the period of our study from 16.26% in 2014 to 16.41% in 2020 with a peak of 17.99% in 2018. This peak of our milieu could be explained by factors described in the literature such as: improvement general operating technique and anesthesia-resuscitation offering more security for the mother-child couple [10, 24], progress in neonatal resuscitation authorizing the extraction of hypotrophic and premature babies with a chance of survival without sequelae neurological [25], the improvement and, for some, the excessive use of electronic monitoring of parturition which would contribute to sometimes abusive caesarean sections [26, 27], the demographic changes that have occurred in developed countries which have reduced the number average number of children per family so that each child becomes more precious and the limitation of the number of births often imposed, rightly or wrongly, by caesarean section ceases to be bad accepted [12] and commercial and convenience motivations [14].

About two out of three caesarean sections were performed in a context of obstetric emergency from the outset, i.e. 65.0% of cases against 40.50% performed after attempted vaginal delivery and 4.50% in a scheduled or elective manner. The place occupied by emergency caesareans in this study is also similar to that of other authors in our country and in other developing regions who report rates of emergency caesareans varying from 58 to 96% and evoke the various reasons, in particular: the absence of real prenatal consultations that can select and prophylactically guide high-risk pregnant women towards specialized structures, the resurgence of makeshift and invalid peripheral maternity wards, the underqualification of staff working in peripheral maternity wards, ignoring contraindications of the vaginal route and only referring pregnant women very late, the pronatalist ethnoculture that characterizes our populations, including most health professionals, devoting the vaginal route as the only happy motherhood, the cesarean section being wrongly deemed to limit births; poor distribution of health centers and difficulties in accessing referral structures; and last but not least, the poverty and illiteracy that characterize our populations. These are the common and characteristic factors of developing countries [11, 12, 14, 19, 20].

In the literature, there are multiple indications for caesarean sections and any obstetric pathology can be one [28]. In our case series, we have the following indications: hemorrhagic placenta previa, foeto-pelvic disproportion, dynamic dystocia complicated by acute fetal distress, uterine rupture, narrowed pelvis, beating cord prolapse, transverse presentation, malposition fetus and other indications. The increase in the indications for caesarean sections in the event of arterial hypertension responds to the recommendations of a consensus which states that a caesarean section is indicated if the hypertension is severe or unstable from the 34th week of amenorrhea. In fact, during this period, the fetus runs a greater risk of dying from hypertension than from prematurity [20]. In our case series, no caesarean section was indicated for hypertension. Regarding HIV infection, it is an indication for elective caesareans for most European authors because it reduces the risk of mother-to-child transmission [29]. In our environment, we opt for vaginal delivery in the absence of obstetric contraindications. However, this vaginal delivery is done under an antiretroviral protocol and precautions must be taken during this delivery to protect the baby and the staff.

The weakness of our study is not to have studied the types of complications of caesarean section at the same time while its strength is to be the first to study the epidemiological particularity and indications of caesarean section in the hospitals of Kananga in the Central Kasai.

## Conclusion

It clearly appeared in this study that the frequency of caesarean section was about one in six parturients delivered by caesarian section, primiparity was most affected, hemorrhagic placenta previa was most frequent indication, its complications presented about half of cases and the maternal mortality rate due to cesarean section was of 531 maternal deaths per 100.000 live births. Our results serve as the basis for in-depth studies on the indications and complications of caesarean section in order to reduce the risks associated with the practice of caesarean section in our environment.

### What is known about this topic

- The purpose of the cesarean section is to save the newborn and the mother in situations where the vital prognosis of the fetus and mother is endangered;
- The frequency of cesarean section has continued to increase in recent decades, but this increase differs enormously from one country to another and in the same environment, from one medical institution to another;
- The lack of data on the epidemiology and indications of caesarean section in hospitals of Kananga, in the DR Congo.

### What this study adds

- The frequency of Cesarean section is about one in six parturients delivered by caesarian section;
- The average age of patients is of 25.39 years, the primiparaous is more concerned, haemorrhagic placenta previa is the most frequent indication, its complications are present in about half of cases, maternal mortality rate linked to caesarean section was of 531 maternal deaths per 100.000 live births;
- Our results are the basis of deepened studies upon intra operative complications of cesarean-section in order to reduce the risk linked to cesarean section´s practice in our milieu.

## References

[1] Sepou A, Yanza MC, Nguembi E, Ngbale R, Kouriah G, Kouabosso A (2000). Etude de 299 cas de césariennes pratiquées à l´hôpital communautaire de Bangui. Méd Afr Noire.

[2] Trabelsi K, Jedidi J, Yaich S, Louati D, Amouri H, Gargouri A (2006). Les complications maternelles peropératoires de la césarienne: Apropos de 1404 cas. JIM Sfax.

[3] Betrán AP, Ye J, Moller AB, Zhang J, Gülmezoglu AM, Torloni MR (2016). The Increasing Trend in Caesarean Section Rates: Global, Regional and National Estimates: 1990-2014. PLoS One.

[4] Vogel JP, Betran AP, Vindevoghel N, Souza JP, Torloni MR, Zhang J (2015). Use of the Robson classification to assess caesarean section trends in 21 countries: a secondary analysis of two WHO multicountry surveys. Lancet Glob Health.

[5] Zwecker P, Azoulay L, Abenhaim HA (2011). Effect of fear of litigation on obstetric care: a nationwide analysis on obstetric practice. Am J Perinatol.

[6] Mi J, Liu F (2014). Rate of caesarean section is alarming in China. Lancet.

[7] Elshani B, Daci A, Gashi S, Lulaj S (2012). The incidence of caesarean sections in the university clinical center of Kosovo. Acta Inform Med.

[8] Ministère du Plan et Macro International 200 Enquête Démographique et de Santé. République Démocratique du Congo 2007 Calverton, Maryland, USA. Ministère du Plan et Macro International.

[9] Ministère du Plan et Suivi de la Mise en oeuvre de la Révolution de la Modernité (MPSMRM) Ministère de la Santé Publique (MSP) et ICF International, 2014. Enquête Démographique et de Santé en République Démocratique du Congo 2013-2014 Rockville, Maryland, USA. MPSMRM, MSP et ICF International.

[10] Villar J, Valladares E, Wojdyla D, Zavaleta N, Carroli G, Velazco A (2006). Caesarean delivery rates and pregnancy outcomes: the 2005 WHO global survey on maternal and perinatal health in Latin American. Lancet.

[11] Chu K, Cortier H, Maldonado F, Mashant T, Ford N, Trelles M (2012). Cesarean section rates and indications in sub-Saharan Africa : a multi-country study from Médecins sans Frontières. PLoS One.

[12] Kizonde K, Kinekinda X, Kimbala J, Kamwenyi K (2006). La cesarienne en milieu Africain: exemple de la maternité centrale Sendwe de Lubumbashi-RD Congo. Med Afr Noire.

[13] Foumane P, Mve Koh Valère, Ze Minkande Jacqueline, Njofang Ngantcha, Dohbit Sama Julius, Mboudou Emile (2014). Facteurs de risque et pronostic des césariennes d'urgence à l'hôpital gynéco-obstétrique et pédiatrique de Yaoundé (Cameroun). Médecine et Santé Tropicales.

[14] Kinenkinda X, Mukuku O, Chenge F, Kakudji P, Banzulu P, Kakoma JB (2017). Césarienne à Lubumbashi, République Démocratique du Congo I: fréquence, indications et mortalité maternelle et périnatale. Pan Afr Med J.

[15] Cavallaro FL, Cresswell JA, França GV, Victora CG, Barros AJ, Ronsmans C (2013). Trends in caesarean delivery by country and wealth quintile: cross-sectional surveys in southern Asia and sub-Saharan Africa. Bull World Health Organ.

[16] Kizonde K, Kalala-Tshibangu C, Kakoma-Sakatolo Z, Kanivo M (2006). Cervicogramme linéaire préfiguratif dans la gestion de la phase active du travail en régions sous-développées impact sur le mode d'accouchement et la mortalité foeto-maternelle: résultats d'une expérimentation en République Démocratique du Congo. Médecine d'Afrique Noire.

[17] Hsia RY, Mbembati NA, Macfarlane S, Kruk ME (2012). Access to emergency and surgical care in sub-Saharan Africa: the infrastructure gap. Health Policy Plan.

[18] Lokomba BV, Kinuka MA (2015). Complications de la césarienne aux Cliniques Universitaires de Kinshasa. Kisangani Médical.

[19] Ouédraogo CM, Ouédraogo A, Ouattara A, Lankoandé J (2015). La pratique de la césarienne dans un hôpital de district à Ouagadougou: aspects épidémiologiques, cliniques et pronostiques: à propos de 3381 cas. Médecine et Santé Tropicales.

[20] Bokossa M, Nguessan K, Doumbia Y, Kakou C, Djougou C, Boni S (2008). Césariennes prophylactiques et d'urgence: à propos de 394 cas au CHU de Cocody (Abidjan). Médecine d'Afrique Noire.

[21] Mbungu MR, Ntela A, Kahindo MP (2015). Classification des césariennes selon Robson à Kinshasa. Kisangani Médical.

[22] Shah A, Fawole B, M´Imunya JM, Amokrane F, Nafiou I, Wolomby JJ (2009). Cesarean delivery outcomes from the WHO global survey on maternal and perinatal health in Africa. Int J Gynaecol Obstet.

[23] Organisation Mondiale de la Santé (2014). Déclaration de l'OMS sur les taux de césarienne. Genève. OMS.

[24] Rozenberg P (2004). L'élévation du taux de césariennes: un progrès nécessaire de l'obstétrique moderne. J Gynecol Obstet Biol Reprod.

[25] O'Driscoll K, Foley M (1983). Correlation of decrease in perinatal mortality and increase in cesarean section rates. Obstet Gynecol.

[26] Haverkamp AD, Orleans M, Langendoerfer S, McFee J, Murphy J, Thompson HE (1979). A controlled trial of the differential effects of intrapartum fetal monitoring. Am J Obstet Gynecol.

[27] Barber EL, Lundsberg L, Belanger K, Pettker CM, Funai EF, Illuzzi JL (2011). Indications Contributing to the Increasing Cesarean Delivery Rate. Obstet Gynecol.

[28] Racinet C, Favier M, Malinas Y (1994). La césarienne : indications téchniques et complicatons Ed Masson. Paris.

[29] Read JS, Newell MK (2005). Efficacy and safety of cesarean delivery for prevention of mother-to-child transmission of HIV-1. Cochrane Database Syst Rev.

